# The effect of the polycystic ovary syndrome and hypothyroidism on the risk of fibrocystic breast changes: a meta-analysis

**DOI:** 10.1186/s12935-022-02547-5

**Published:** 2022-03-19

**Authors:** Parisa Kohnepoushi, Hojat Dehghanbanadaki, Pardis Mohammadzedeh, Maziar Nikouei, Yousef Moradi

**Affiliations:** 1 Student Research Committee, Kurdistan University of Medical Sciences, Sanandaj, Iran; 2 Students Scientific Research Center, Tehran University of Medical Sciences, Tehran, Iran; 3 Department of Epidemiology and Biostatistics, Faculty of Medicine, Kurdistan University of Medical Sciences, Sanandaj, Iran; 4 Social Determinants of Health Research Center, Research Institute for Health Development, Kurdistan University of Medical Sciences, Sanandaj, Iran

**Keywords:** Polycystic ovary syndrome, Hypothyroidism, Fibrocystic change, Fibrocystic breast disease, Meta-analysis

## Abstract

This meta-analysis aimed to determine the pooled association between polycystic ovary syndrome (PCOS), hypothyroidism, and fibrocystic breast changes. We searched important databases, including PubMed (Medline), Scopus, Web of Science, and Embase to retrieve all relevant studies published from 1990 to April 2021. The bias risk of selected articles was assessed based on the JBI checklist. Our search strategy yielded a total of 487 articles from the international databases. After screening their full-texts, 6 articles met the inclusion criteria and were considered for meta-analysis. The effect of PCOS on the incidence of fibrocystic breast changes was 2.49 (95% CI 1.85–3.34). Also, the effect of hypothyroidism on the incidence of fibrocystic breast changes was 1.90 (95% CI 0.92–3.93). The results showed that women with PCOS were at higher risks to develop fibrocystic breast changes.

## Introduction

Polycystic ovary syndrome (PCOS) is the most common hormonal disorder of women in the childbearing age and the leading cause of hyperandrogenism and ovulation disorders which cause impaired fertility [1, 2]. The prevalence of this disease based on different diagnostic criteria, including the ones of the National Institutes of Health (NIH), the Rotterdam, and the Androgen Excess and PCOS Society (AES), is estimated between 9 and 18%, the highest rate of which belongs to Western societies [1, 3–5]. Clinical symptoms of this syndrome include hirsutism, acne, and alopecia [6, 7]. Patients may also experience irregular bleeding and infertility. Patients with PCOS are at risk for a wide range of endocrine and metabolic disorders, including insulin resistance (IR) and the metabolic syndrome. This population is also at an increased risk of acanthosis nigricans, type 2 diabetes mellitus, dyslipidemia, visceral obesity, cardiovascular diseases, and endometrial cancer [1, 8, 9]. There are three types of diagnostic systems for PCOS: the NIH guideline, the Rotterdam system, and the AES guideline. In 1990, the NIH guideline was proposed for the diagnosis of PCOS with two criteria of oligo/amenorrhea and clinical/laboratory findings of hyperandrogenism [10]. In 2003, the Rotterdam system defined PCOS as the presence of two out of three criteria of clinical/laboratory evidence of hyperandrogenism, oligo/amenorrhea, and the polycystic ovaries on sonography [11, 12]. Later in 2006, the AES guideline was developed for PCOS diagnosis. In this guideline, PCOS was defined as the presence of hyperandrogenism and one or both features of oligo/amenorrhea and polycystic ovaries on sonography [12, 13]. Breast complaints account for a significant proportion of women's health problems with a prevalence of 16% to 50% in various reports. Studies have shown that approximately half of women who visit clinics with breast-related symptoms are affected by benign breast disorders, e.g., fibrocystic changes which occur in 50% of patients over the age of 30 years. Fibrocystic breast changes are the most common benign breast changes, found in 50% of women undergoing clinical examination and 90% of women undergoing histopathological examination. Fibrocystic breast changes are common in women aged 20 to 50 years [14, 15] and their symptoms such as nipple pain and discharge can adversely affect the life quality of premenopausal women. In some previous reports, the significant association between fibrocystic breast changes and PCOS has been reported while in some others, no association has been established [16–19]. Thyroid disorders are one of the most common endocrine disorders more common in women than men. Although the cause of benign breast disorders, especially fibrocystic changes, is unknown, endocrine disorders with decreased thyroid function have been shown to associate with these changes. So far, various studies have been performed to determine the association of PCOS and thyroid disorders with fibrocystic breast changes in women around the world, but on the one hand, the number of these studies is very small and on the other hand, contradictory results have been reported from these studies [16–21]. Therefore, in this research, we reviewed the literature to determine the pooled relationship between PCOS, hypothyroidism, and fibrocystic breast changes.

## Materials and methods

In this study, we followed 13 steps to perform meta-analysis [22]; we determined the research question with the PECOS template, conducted a preliminary search, determined the inclusion and exclusion criteria, wrote a search strategy, searched four international databases, screened the retrieved articles based on their titles and abstracts, performed full-text screening, conducted a manual search for additional relevant studies, extracted data from included studies and performed the quality assessment, checked the extracted data, performed the statistical analysis (Meta-analysis), double-checked the data, and finally, we wrote the manuscript, revised it, and submitted it. This meta-analysis was in accordance with the guideline of the Preferred Reporting Items for Systematic Reviews and Meta-analyses (PRISMA) [23, 24].

### Search strategy and screening

We conducted this study to answer the following research question: “What is the relative risk of the fibrocystic breast disease with polycystic ovary syndrome and hypothyroidism among women?” In this study, first, the main keywords were determined based on the research question and the purpose of the study. The main keywords included hypothyroidism, polycystic ovary syndrome, and fibrocystic breast changes. Besides, the research question was based on the structure of PECOS (Population, Exposure, Comparison, Outcomes, and the Type of study) and the search strategy was based on exposure and outcomes, and it respectively included hypothyroidism, PCOS, and fibrocystic breast changes compiled and implemented together with their synonyms in international databases. Databases searched in this study included PubMed (Medline), Scopus, Web of Science, and Embase. The search was conducted from 1990 to April 2021. Related gray articles were also searched in google scholar. Moreover, references of related articles were finally reviewed and related articles were also included.

### Eligibility criteria

In this meta-analysis, articles whose main purpose was to investigate the pooled association of hypothyroidism or PCOS with fibrocystic breast changes were included. Case–control or cohort studies in which the study population covered women with hypothyroidism or PCOS were analyzed to determine the relative risk of fibrocystic breast changes. In this case, we included all studies which had identified their PCOS patients with either the NIH criteria, Rotterdam criteria, or AES criteria. Review studies, reports, case reports, case series, letters to the editor, and clinical trials were excluded. Also, studies whose full texts were not available, first, were attempted to be got by sending an email to their authors in charge, and if the corresponding authors did not respond, these studies would be excluded from the analysis.

### Screening and data extraction

Articles were screened based on their titles, abstracts, and full texts after completing the search strategy in different databases and removing duplicates. After screening, the data extraction form was designed using the opinions of experts in this field, e.g., obstetricians and epidemiologists. The data extraction form included (1) the first author’s name, (2) the article publication year, (3) the country, (4) controlled variables, (5) age, (6) information about people with fibrocystic breast changes, (7) the study population and (8) diagnosis methods of fibrocystic breast changes, PCOS, and hypothyroidism.

All stages of article screening, as well as data extraction were independently performed by the two authors. If there was a dispute in these cases, it was resolved with the opinion of a third expert.

### Quality assessment or risk of bias

In this study, quality assessment of selected articles was performed based on the JBI checklist. This checklist is used to assess the risk of bias in case–control and cohort studies and has 10 questions about how to sample, select groups, compare outcomes, and measure them. Finally, the articles were classified as Low, Moderate, and High from the viewpoint of quality. All steps of quality evaluation of selected articles were independently performed by the two authors. If there was a dispute in this case, it would be resolved with the opinion of a third expert.

### Statistical analysis

The analysis indicators included the odds ratio and risk ratio along with their 95% confidence interval (95% CI) reported in the case–control and cohort studies, respectively. The analysis model was a random-effects or fixed-effects model (taking into account the conditions). In this meta-analysis, because the prevalence of fibrocystic breast changes in women was less than 0.05 according to the previous reports, the study outcome was considered rare and the association index was considered equal to the relative risk by the combination of the odds ratio in case–control studies and the risk ratio in cohort studies.

The percentage of heterogeneity in this study was expressed using I square and Q Cochrane index. According to the reported criteria of Q Cochrane, 0% to 25% indicates no heterogeneity, 25% to 50% low heterogeneity, 50% to 75% high but acceptable heterogeneity, and 75% to 100% high and unacceptable heterogeneity. The publication bias was assessed using the Eggers test. Because the number of included articles was less than 10, the Funnel Plot was not reported. Subgroup analyzes were not performed in this meta-analysis due to the small number of studies and the lack of reporting important variables in the studies selected for analysis. All analyzes were performed in STATA software version 16.

## Results

### Qualitative results

**Fig. 1:**
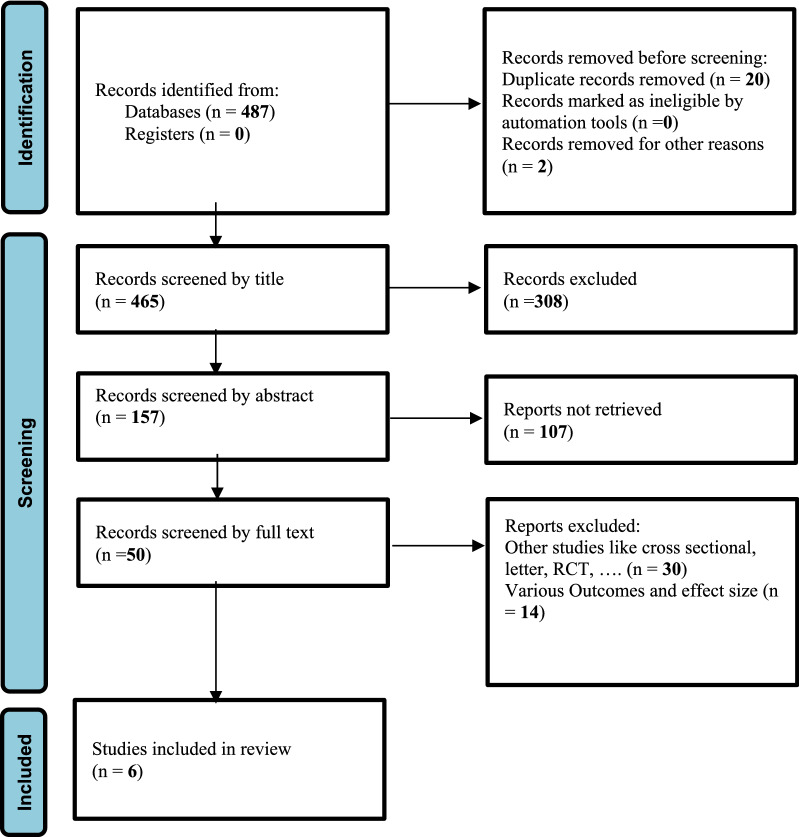
PRISMA flow diagram which included searches of databases and registers only

Our search strategy yielded a total of 487 articles from the international databases. After removing duplicates, 465 articles were screened based on their titles. Then, 157 were screened based on their abstracts, and finally, after removing 107 articles, 50 studies were screened based on their full texts. At the end, 6 articles [16–21] which met the inclusion criteria, were considered for meta-analysis (Fig. 1). The general characteristics of the studies included in this meta-analysis were recorded in Table 1, which consisted of 5 case–control studies [16, 18–21] and one cohort study [17]. A total of 1533 participants in the age range of 17 to 71 years, were included in the study. Two studies [20, 21] with 487 participants examined the effect of hypothyroidism on fibrocystic breast changes, one [21] of which assessed the prevalence of fibrocystic breast changes in patients with Hashimoto's thyroiditis, an autoimmune thyroid disease and a major cause of hypothyroidism in areas with sufficient iodine. In other 4 studies [16–19] with 1046 participants, the impact of PCOS on fibrocystic breast changes was investigated. These studies were conducted in different countries such as Turkey [16, 18, 21], Iran [20], the USA [17], and Italy [19] between 2001 and 2020.

**Table 1:** The characteristic of included case–control and cohort studies in this meta-analysis

Authors (Year)	Study population	Age (year)	Sample size	Fibrocystic changes of breast detection	PCOs detection	Hypothyroidism detection	Measurement of association, relative risk (CI 95%)	Controlled variables
Alipour et al. [20] (2018)	Women with fibrocystic changes (FCC) and women who had attended the clinic for breast cancer screening without any breast symptoms	Case = 40 ± 8.8 Control = 46 ± 9.8	320 Case = 114 Control = 206	Breast clinical features or ultrasonographic and histological detection of FCC		TSH ≥ 5 (μIU/mL)	1.52 (0.61, 3.78)	Age, body mass index (BMI), and family history of BBD
Anil et al. [21] (2015)	Patients with HT, subjects without known thyroid disease, who attended the Endocrinology Department for general Endocrine check-up	Case = 37.66 ± 8.41 Control = 32.59 ± 7.32 (18_50)	167 Case = 95 Control = 72	Breast ultrasonography		Thyroid ultrasonography, positive anti-TPO (antithyroid peroxidase)	2.81 (0.84, 9.40)	
Gungor et al. [16] (2020)	Women with Irregular Menstruation, and Polycystic Ovary Syndrome, who applied to the Gynecology and Obstetrics outpatient clinic	Case = 33.02 ± 1.99 Control = 33.2 ± 1.9	269 Case = 102 Control = 167	Breast ultrasound	Based on the Rotterdam 2003 diagnostic criteria		1.41 (0.86, 2.31)	Age _BMI Mean age at menarche _smoking
Soran et al. [17] (2005)	Women with PCOS and healthy from the population of a cohort study	Case = 46 (30–66) Control = 47 (29–63)	240 Case = 116 Control = 124	Clinical or histological diagnosis	Through private reproductive endocrine practices		0.78 (0.45, 1.36)	Age, Race
Gumus et al. [18] (2009)	PCOS and non- PCOS	Case = 25 ± 4.98 Control = 26.4 ± 5.09	93 Case = 53 Control = 40	Ultrasound examination	Laboratory, clinical, and ultrasound findings		3.17 (1.31–7.68)	Age, body mass Index (BMI)
D’Amelio et al. [19] (2001)	Women who attend the outpatient ultrasound clinic	26 ± 1 (18–30)	444 Case = 93 Control = 351	Ultrasound mammography	Pelvic ultrasound scan		18.05 (10.07, 32.35)	Age, parity, menstrual patterns, body mass index (BMI)

These studies used mammography, sonography, and histological evaluation to diagnose fibrocystic breast changes, as well as laboratory, clinical and sonography findings to diagnose PCOS, and TSH level assessment, thyroid sonography, and antithyroid peroxidase (anti-TPO) assessment to diagnose hypothyroidism.

### Quantitative results

#### Impact of PCOS on the development of fibrocystic breast changes

**Fig. 2:**
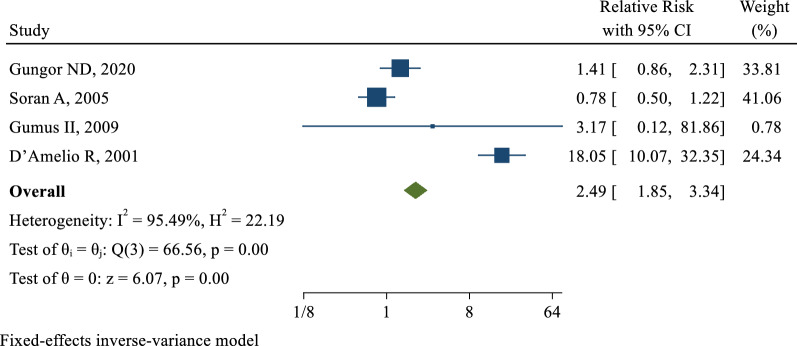
The pooled effect of Polycystic Ovary Syndrome on the risk of Fibrocystic Disease of Breast

4 [16–19] of the 6 included studies examined the effect of PCOS on the incidence of fibrocystic breast changes. The risk ratio in these studies was in the range of 0.78 (95% CI 0.45–1.36) to 18.05 (95% CI 10.07–32.35). After combining these studies, the pooled relative risk was 2.49 with a confidence interval of 1.85 to 3.34. This result showed that the risk of developing fibrocystic breast changes increased by approximately 2.5 times in women with PCOS compared to those without PCOS (Fig. 2). The publication bias was examined by the egger's test, which showed that the publication bias did not occur in these studies (B: 0.76; SE: 0.09; P: 0.339).

#### Impact of hypothyroidism on the development of fibrocystic breast changes

**Fig. 3:**
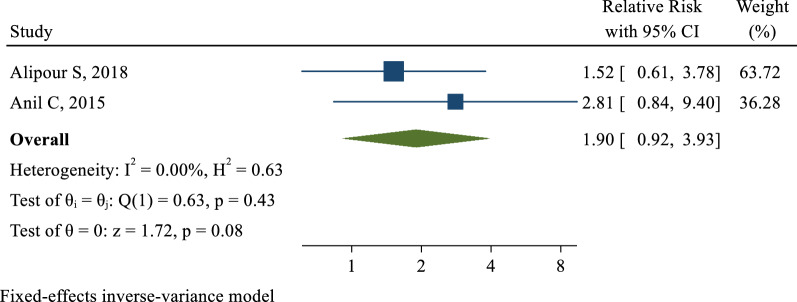
The pooled effect of Hypothyroidism on the risk of Fibrocystic Disease of Breast

2 [20, 21] of the 6 included studies examined the effect of hypothyroidism on the incidence of fibrocystic breast changes. The association range measured in these studies was from 1.52 (95% CI 0.61–3.78) to 2.81 (95% CI 0.84–9.40). After combining the results, the pooled relative risk was 1.90 with a confidence interval of 0.92 to 3.93. Therefore, the risk of developing fibrocystic breast changes in women with hypothyroidism was 1.90 times higher than women without this condition, but this effect was not statistically significant (Fig. 3).

## Discussion

The results of our study showed that there was a statistically significant association between fibrocystic breast changes and PCOS as well as people with PCOS had a higher chance of developing fibrocystic breast changes than people without this syndrome. The positive relationship between PCOS and fibrocystic breast changes can be attributed to several mechanisms. Because there is a distinct degree of hyperandrogenism in PCOS, this hyperandrogenism can exert inhibitory effects on progesterone and consequently leads to increase mammary epithelial cell proliferation, breast growth, and fibrocystic breast formation [25–29]. The next mechanism is the possibility of converting androgens to estrogen and the stimulatory effects of estrogen on the growth and division of the mammary epithelium [30–34]. Besides, the lack of ovulation in patients with PCOS can be considered as the next mechanism. Gumus et al. in their case–control study showed that out of 53 PCOS cases, 21 had fibrocystic breast change characteristics (39.6%) while out of 40 healthy controls, not suffering from PCOS, only 4 had fibrocystic breast changes (12.5%) and this difference between two groups was statistically significant [18]. In another case–control study, D'Amelio et al. examined the association between PCOS and fibrocystic breast changes. Participants of this study were a group with PCOS and a healthy control group without PCOS. The prevalence of fibrocystic breast changes in case and control groups was 92% and 7%, respectively, which showed a significant relationship between these two diseases [19]. However, we should notice that the number of people in the case group was very low. The results of these studies were in line with our pooled results while different findings were reported in the study performed by Soran et al. Participants in this cohort study included 116 patients with PCOS as the case group and 114 healthy individuals without PCOS as the control group. The two groups were age-matched, and the criteria for diagnosing PCOS for the case group included chronic anovulation, clinical symptoms of hyperandrogenism, free testosterone levels greater than 2 nmol/L, and the LH to FSH ratio greater than 2. At the end of this study, the fibrocystic breast disease was found in 19 patients in the case group (16%) and 26 patients in the control group (21%). The prevalence of the fibrocystic breast disease was lower than expected in the case group and as a result, no clear association was found between PCOS and the fibrocystic breast disease [17].

In another part of our study, a weak positive relationship was found between hypothyroidism and fibrocystic breast changes. So, people with hypothyroidism were more likely to develop the fibrocystic breast disease. However, this association was not statistically significant. Various mechanisms have been proposed for the effect of hypothyroidism on breast cells. One mechanism is that low levels of thyroid hormones in the body suppress negative self-regulation of the pituitary-hypothalamic axis, resulting in increased secretion of prolactin-like thyroid-stimulating hormone (TSH) which can affect breast cells and cause changes in breast parenchyma [14, 20, 21, 35]. The next mechanism suggests that lower than normal levels of thyroid hormones may abnormally increase the sensitivity of breast epithelial cells to the stimulatory effect of prolactin [36–38]. As a result, it can cause metaplasia and even dysplasia of the breast tissue [35]. However, the cause of this possible relationship between thyroid disorders and the breast disease is still unclear. Falstie-Jensen et al. in a cohort study on the association between hypothyroidism and the fibrocystic breast disease, examined 35,463 women with non-metastatic breast cancer, who were diagnosed with cancer between 1996 and 2009. Initially, 1272 patients with breast cancer (4%) had hypothyroidism and the remaining 34,191 patients (96%) had healthy thyroid. Then, patients with healthy thyroid were followed up for an average of 6 years. After a mean period of 3.4 years, 859 patients (2%) out of 34,191 ones with healthy thyroid developed hypothyroidism. Nevertheless, no significant relationship was reported between hypothyroidism and the fibrocystic breast disease (either at the time of diagnosis or during follow-up of patients) [39]. Anil et al. designed a case–control study to evaluate the prevalence of benign breast diseases in patients with thyroid disorders. The study groups included a group of 71 patients with nodular goiter (NTD) and a group of 95 patients with Hashimoto's thyroiditis (HT) as the case groups and 72 individuals with normal thyroid hormones who did not have any underlying disease as the control group [21]. At the end of this study, 8 (11.3%) NTD patients, 11 (11.6%) HT patients, and 4 (5.6%) healthy controls had fibrocystic changes. Although the prevalence of fibrocystic changes was higher in the case groups, this higher prevalence did not show a statistically significant association between hypothyroidism and fibrocystic breast changes [21]. Kuijpens et al. examined the relationship between hypothyroidism and breast cancer in a cross-sectional and prospective design. The target group consisted of 2775 women randomly selected from 8503 participants in the EPOS study. In the initial study, 37 women (1.3%) had breast cancer. The levels of free TSH and T4 hormones in these patients were statistically the same as in those without breast cancer. Then, in the prospective part of the study, the remaining 2738 women were followed up. After excluding 230 women due to various reasons, 61 women (2.2%) developed breast cancer over 9 years. These people had lower levels of free T4 compared to women without breast cancer. This difference was statistically significant (OR = 2.3) and lower levels of T4 were suggested as an independent risk factor for breast cancer [40].

One of the strengths of this study is the importance of this issue in clinical practice and the provision of preventive services for women with PCOS and hypothyroidism. The main limitations can be mentioned as the small number of studies included in the analysis and the lack of subgroup analyzes based on important and affective variables such as the age, statistical population, history of disorders, and important diseases regarding fibrocystic breast changes. Thus, we require further large-scale cohort studies on the population with PCOS, and hypothyroidism together with healthy controls matching in age and other comorbidities to confirm our results.

## Conclusion

PCOS women are at higher risks to develop fibrocystic breast changes. Thus, for women with this syndrome, the necessary screening and counseling should be provided as regards the incidence of fibrocystic breast changes. In addition, planning for early diagnostic procedures of fibrocystic changes can be considered in these communities.

## Data Availability

Not applicable.

## Abbreviations

PCOS: Polycystic ovary syndrome
OR: Odds ratio
CI: Confidence interval
TSH: Thyroid-stimulating hormone
HT: Hashimoto's thyroiditis
Anti-TPO: Antithyroid peroxidase
PRISMA: Preferred Reporting Items for Systematic Reviews and Meta-Analyzes
